# Characterizing Chemoautotrophy and Heterotrophy in Marine Archaea and Bacteria With Single-Cell Multi-isotope NanoSIP

**DOI:** 10.3389/fmicb.2019.02682

**Published:** 2019-12-17

**Authors:** Anne E. Dekas, Alma E. Parada, Xavier Mayali, Jed A. Fuhrman, Jessica Wollard, Peter K. Weber, Jennifer Pett-Ridge

**Affiliations:** ^1^Department of Earth System Science, Stanford University, Stanford, CA, United States; ^2^Physical and Life Sciences Directorate, Lawrence Livermore National Laboratory, Livermore, CA, United States; ^3^Department of Biological Sciences, University of Southern California, Los Angeles, CA, United States

**Keywords:** Thaumarchaeota, marine archaea, nanoSIMS, chemoautotrophy, single cell, stable isotope probing, ^13^C, ^15^N

## Abstract

Characterizing and quantifying *in situ* metabolisms remains both a central goal and challenge for environmental microbiology. Here, we used a single-cell, multi-isotope approach to investigate the anabolic activity of marine microorganisms, with an emphasis on natural populations of Thaumarchaeota. After incubating coastal Pacific Ocean water with ^13^C-bicarbonate and ^15^N-amino acids, we used nanoscale secondary ion mass spectrometry (nanoSIMS) to isotopically screen 1,501 individual cells, and 16S rRNA amplicon sequencing to assess community composition. We established isotopic enrichment thresholds for activity and metabolic classification, and with these determined the percentage of anabolically active cells, the distribution of activity across the whole community, and the metabolic lifestyle—chemoautotrophic or heterotrophic—of each cell. Most cells (>90%) were anabolically active during the incubation, and 4–17% were chemoautotrophic. When we inhibited bacteria with antibiotics, the fraction of chemoautotrophic cells detected *via* nanoSIMS increased, suggesting archaea dominated chemoautotrophy. With fluorescence *in situ* hybridization coupled to nanoSIMS (FISH-nanoSIMS), we confirmed that most Thaumarchaeota were living chemoautotrophically, while bacteria were not. FISH-nanoSIMS analysis of cells incubated with dual-labeled (^13^C,^15^N-) amino acids revealed that most Thaumarchaeota cells assimilated amino-acid-derived nitrogen but not carbon, while bacteria assimilated both. This indicates that some Thaumarchaeota do not assimilate intact amino acids, suggesting intra-phylum heterogeneity in organic carbon utilization, and potentially their use of amino acids for nitrification. Together, our results demonstrate the utility of multi-isotope nanoSIMS analysis for high-throughput metabolic screening, and shed light on the activity and metabolism of uncultured marine archaea and bacteria.

## Introduction

Despite recent advances in our understanding of environmental microbiology due to next generation sequencing, characterizing and quantifying the activity of microorganisms in the environment remains a challenge. Fundamental questions such as what percentage of cells is active/inactive and autotrophic/heterotrophic within a natural community remain unanswered. These questions require culture independent, quantitative, single-cell approaches. Techniques to accomplish this exist, such as nanoSIP (Pett-Ridge and Weber, 2012) and microautoradiography (Okabe et al., 2004), and illustrate within-population variation in metabolism and growth (e.g., Popa et al., 2007; Finzi-Hart et al., 2009; Kopf et al., 2015; Zimmermann et al., 2015; Dawson et al., 2016; Dekas et al., 2016). However, these studies are often limited in scope due to their low-throughput, typically analyzing a few to dozens of cells per analysis.

Oceans comprise one of the largest microbial habitats on earth, and marine microorganisms play central roles in global biogeochemical cycling. However, the marine microbial community is one of the least well-represented in culture collections (Lloyd et al., 2018), and the activity and function of many of its members are unknown. One of the most abundant microbial groups in the ocean is the Marine Group I (MGI) Thaumarchaeota (Karner et al., 2001), recently proposed to be reclassified as within the Nitrososphaeria, a class within the Crenarchaeota (Parks et al., 2018). They have largely evaded isolation, and only a handful of representatives have been investigated in laboratory culture or enrichments (Konneke et al., 2005; Santoro and Casciotti, 2011; Mosier et al., 2012; Berg et al., 2014; Qin et al., 2014; Ahlgren et al., 2017). Multiple lines of evidence suggest that marine Thaumarchaeota are chemoautotrophic, coupling inorganic carbon fixation to the oxidation of ammonia to nitrite (Pearson et al., 2001; Herndl et al., 2005; Konneke et al., 2005; Hallam et al., 2006; Beman et al., 2008). However, environmental studies suggest that this group obtains at least a fraction of its carbon from organic sources (Ouverney and Fuhrman, 2000; Teira et al., 2004; Herndl et al., 2005; Ingalls et al., 2006). These studies used single-isotope and/or bulk analysis methods, and were therefore unable to discern if individual Thaumarchaeota are capable of both inorganic and organic carbon assimilation (mixotrophy), or if intra-phylum variability in carbon assimilatory capabilities exists.

To characterize the activity and metabolism of natural populations of marine bacteria and archaea, including Thaumarchaeota, we developed an analysis protocol where microorganisms incubated with ^13^C-bicarbonate and ^15^N-amino acids and analyzed by nanoscale secondary ion mass spectrometry (nanoSIMS) could be rapidly and individually identified as active/inactive and autotrophic/heterotrophic. Using this approach, we analyzed 1,501 individual marine microorganisms collected in the Pacific Ocean coastal euphotic and aphotic zones (0 and 150 m water depth, respectively). We determined: (1) the fraction of anabolically active cells, (2) the range of anabolic activity within the community, and (3) the fraction of chemoautotrophic and heterotrophic cells in each sample. To specifically investigate the activity of the Thaumarchaeota, we measured differences in single-cell metabolic activity profiles with and without bacterial antibiotics (added to enrich for archaea), and used Thaumarchaeota-targeted fluorescence *in situ* hybridization (FISH) coupled to nanoSIMS analysis. We also used amplicon sequencing of partial 16S rRNA genes and 16S rRNA to characterize the microbial community throughout the incubation experiments. To summarize, we developed a high-throughput nanoSIMS approach to screen the metabolic activity of individual, unidentified cells, and employed it as well as DNA sequencing and FISH-nanoSIMS to establish a greater understanding of uncultured marine microorganisms.

## Materials and Methods

### Sample Collection

Samples were collected at two sites in the Pacific Ocean: (1) 17 km off the coast of Los Angeles in the San Pedro Basin at the site of the San Pedro Ocean Time Series (SPOT; 33° 33′ 00″, −118° 24′ 00″), and (2) 0.35 km off the coast of western San Francisco at the end of Pacifica Pier (PP; 37° 38′ 00″, −122° 29′ 53″) (Supplementary Figure S1). A Niskin rosette on board the R/V Yellowfin was used to collect the SPOT samples at 150 m water depth on November 13, 2013. Environmental data at the time of sampling is available in Supplementary Table S1. A line and bucket was used to collect the Pacifica samples from the surface on October 5, 2013. Water was stored in polypropylene cubitainers or polycarbonate carboys wrapped in opaque bags, and transported back to the laboratory on ice. Additionally, at SPOT, approximately 10 L of water were filtered on board onto 0.2 μm polyethersulfone Sterivex filters and stored in liquid nitrogen for nucleic acid extraction.

### Bottle Experiments

Seawater from both sites was incubated in 11.3 L polycarbonate carboys in the dark at 9–10°C, close to the *in situ* values of 9.8°C at SPOT and 13°C at Pacifica. Incubations were amended with both ^13^C-bicarbonate (Cambridge Isotopes CLM-441-5; final calculated concentration and isotopic composition of 2.62 mM total bicarbonate at 13.1 at% ^13^C at SPOT, and 2.46 mM total bicarbonate at 7.5 at% ^13^C at Pacifica, assuming natural bicarbonate concentrations of 2.3 mM) and ^15^N-amino acids (Cambridge Isotopes NLM-2161-1; final calculated concentration and isotopic composition of 50 nM total amino acids at 98 at% ^15^N at SPOT, and 100 nM total amino acids at 98 at% ^15^N at Pacifica, assuming natural free amino acids were negligible at both sites). At Pacifica, additional incubations were amended with dual-labeled ^13^C^15^N-amino acids (Cambridge Isotopes CNLM-452-1; final calculated concentration and isotopic composition of 104 nM at 98 at% ^13^C and ^15^N, again assuming natural free amino acids were negligible). Note, the amino acid mixtures lacked glutamine, asparagine, cysteine, and tryptophan; more information about the content of the mixture is available on the manufacturer’s website. A subset of the ^13^C-bicarbonate and ^15^N-amino acid incubations from both sites were amended with the bacterial antibiotics ampicillin sodium salt and streptomycin sulfate salt (0.05 mg/ml final concentration of each). The incubations were harvested at 2.5 and 7 days (SPOT) and 3 and 6 days (Pacifica). Additional incubation details and a summary of subsequent analyses can be found in Supplementary Table S2.

From each incubation, seawater sub-samples were preserved as follows: (1) 100 ml fixed with 2% paraformaldehyde overnight at 4°C, filtered onto 0.2 μm polycarbonate filters, washed with phosphate buffered saline and ethanol, and frozen at −20°C (for microscopy and nanoSIMS analysis) and (2) ~10 L filtered onto 0.2 μm polyethersulfone Sterivex filters and flash frozen in liquid nitrogen before storage at −80°C (for DNA and RNA analysis).

### Cell Enumeration

Fixed cells filtered on polycarbonate filters were visualized and quantified using DAPI-based Vectashield (cat#H-1200, Vector Laboratories, Burlingame, CA, USA) using a Leica DM5500B microscope with a 100× magnification oil immersion lens. Five areas of each filter were photographed using MetaMorph software version 7.7.3.0 and the DAPI stained cells in each region were manually counted. Using the dimensions of each photographed area, the size of the original filter, and the volume of seawater filtered through each filter, an average cell count was calculated for each ml of seawater collected. For samples with high standard deviations after the first five areas were counted, cells in an additional five areas were enumerated.

### Nucleic Acid Extraction

DNA and RNA was extracted from frozen filters using the AllPrep DNA/RNA mini kit (cat# 80204, Qiagen, Valencia, CA, USA), modified to include a bead beating step of 2× 15 s at speed 5.5 m/s on a FastPrep instrument using lysing matrix E bead beat tubes (cat# MP116914100, Thermo Fisher Scientific, Waltham, MA, USA) containing 600 μl of AllPrep RLT buffer plus 1% betamercaptoethanol. The RNA elutions were treated to remove co-extracted DNA using 2 units of Turbo DNase (cat#AM1907, Thermo Fisher Scientific, Waltham, MA, USA) and subsequently converted to cDNA using the SuperScript III First Strand RT PCR kit using the manufacturer supplied random hexamers (cat# 18080-400, Thermo Fisher Scientific, Waltham, MA, USA). Successful elimination of co-extracted DNA in the RNA extract was confirmed by lack of PCR amplification of the DNase-treated RNA, as visualized in an agarose Egel Ex (cat# G4020-02, Thermo Fisher Scientific, Waltham, MA, USA).

### DNA and cDNA Illumina Amplicon Sequencing and Analysis

The 16S rRNA gene was amplified from DNA and cDNA using universal PCR primers targeting the V4 and V5 regions, 515F and 926R (Parada et al., 2016), modified with an Illumina adaptor sequence and dual barcodes. DNA and cDNA samples were amplified in triplicate using 5 PRIME HotMasterMix (cat# FP2200400, Thermo Fisher Scientific, Waltham, MA, USA). Each reaction was subjected to 2 min at 95°C, 25 cycles of 95°C for 45 s, 50°C for 45 s, and 68°C for 90 s, and 78°C for 300 s. Control reactions containing an even mock community, a staggered mock community, and a negative control without DNA were also amplified (as described in Parada et al., 2016). The triplicate amplification products were combined and purified using AGENCOURT® AMPURE® XP beads (cat # A63881, Beckman Coulter, Mountain View, CA, USA) with a 1:1 ratio of bead solution to sample volume and a magnetic separator (A010027, Thermo Fisher Scientific, Waltham, MA, USA). All samples were then pooled in equal volumes and purified again using SPRIselect beads (Beckman Coulter B23317) with a 0.8:1 bead solution to sample volume ratio. Illumina tag sequencing was performed at the UC Davis DNA Technologies Core Facility using a MiSeq (PE300).

Sequences were trimmed using PRINSEQ v. 0.20.4 (Schmieder and Edwards, 2011) and merged with USEARCH10 (Edgar, 2010) allowing a maximum of five mismatches in the overlapping region and a minimum merged length of 250 bp. Merged sequences were demultiplexed, quality filtered, and trimmed of primer sequences in QIIME v1.9 (Caporaso et al., 2010). Sequences were then clustered with Swarm v1.2.19 (Mahe et al., 2014) in QIIME with a resolution of one difference. Representative sequences were assigned taxonomy in QIIME using UCLUST (Edgar, 2010) and the Silva v132 reference dataset (Quast et al., 2013). Any sample with fewer than 1,000 sequences (final range: 1,012–35,065 sequences per sample) and operational taxonomic units (OTUs) with fewer than five total sequences in the dataset was discarded. The relative abundance of all OTUs was calculated as the percentage of sequences assigned to an OTU from the total sequences in a sample, unless otherwise indicated. To determine the variability between sample replicates, the Bray-Curtis Dissimilarity values were determined for all pairs of replicate mock communities, PCR replicates, as well as extraction and elution replicates (Supplementary Table S3) using the “phyloseq” package (McMurdie and Holmes, 2013) and the corresponding similarity values calculated in R (R_Core_Team, 2008). All technical replicates were then averaged with phyloseq and community profiles plotted with the R package ggplot2 (Wickham, 2016). All commands used are available through FigShare 10.6084/m9.figshare.7607423. The sequences have been deposited in the European Molecular Biology Laboratory database under bioproject PRJEB31502.

Microbial community similarities between controls, different treatments, and time points were visualized by non-metric multidimemsional scaling (NMDS) ordination of the Bray–Curtis Dissimilarity between samples using the R packages phyloseq (McMurdie and Holmes, 2013) and ggplot2 (Wickham, 2016). To determine the phylogeny of the most abundant MGI OTUs at Pacifica and SPOT, the representative (most abundant) sequence from each of the 20 most abundant OTUs was aligned with reference sequences using ClustalW in MEGA 6 (Thompson et al., 1994; Tamura et al., 2013). Sequences were trimmed to include only the overlapping regions (382 bp) and a maximum likelihood phylogenetic tree was constructed from the aligned sequences based on the Tamura-Nei model in MEGA6 (Tamura and Nei, 1993).

### Catalyzed Reporter Deposition Fluorescence *in situ* Hybridization

Catalyzed reporter deposition fluorescence *in situ* hybridization (CARD-FISH) was used to identify Thaumarchaeal cells. CARD-FISH was carried out on fixed cells on a polycarbonate filter without embedding according to (Pernthaler et al., 2002; Teira et al., 2004; Pernthaler and Pernthaler, 2007), using the Cren537 probe to target Thaumarchaeota (Teira et al., 2004) and the Eub338I probe to target bacteria (Amann et al., 1990). Permeability was achieved with 2 μg/ml proteinase K from Tritirachium album (EMD Chemicals) in 10× TE buffer for 60 min at 37°C, and hybridization was performed at 20% formamide for 15 h at 35°C (Cren537) and 35% formamide for 4 h at 46°C (Eub338I). Commercially supplied tyramides conjugated to Oregon Green 488 and Alexa 555 were used during the amplification (ThermoFisher Scientific). Samples were treated with hydrogen peroxide (0.1% in PBS for 10 min at 25°C) between serial hybridizations. Negative (no probe) controls did not show amplification signal.

Hybridized cells were mapped and prepared for nanoSIMS analysis according to (Dekas and Orphan, 2011). Briefly, hybridized cells were transferred from the filters to ITO-coated slides by freezing moist filter wedges onto the slides using dry ice, then peeling back the filter. The cells were then counterstained with Vectashield DAPI Mounting Media (Vector Laboratories) and mapped relative to fiducial points on a Leica DM5500B microscope running MetaMorph software with a 100× magnification oil immersion lens. The Vectashield Mounting Media was removed in a water bath before storage and nanoSIMS analysis.

### Nanoscale Secondary Ion Mass Spectrometry

The isotopic composition of individual cells was measured using the CAMECA NanoSIMS 50 housed in the Physical and Life Sciences Directorate at Lawrence Livermore National Laboratory. Fixed cells filtered onto 0.2 μm polycarbonate filters were gold-coated and scanned in a high-throughput manner *via* an automated chain analysis of twelve to sixteen 20 × 20 μm images per sample. To specifically observe Thaumarchaeota, cells hybridized with the Cren537 probe were analyzed during targeted image analyses on ITO-coated glass slides, prepared as described above. A Cs^+^ primary ion beam (2–4 pA) with a nominal spot size of 100–200 nm was used to raster over the cells. Four masses were collected: ^12^C_2_^−^, ^12^C^13^C^−^, ^12^C^14^N^−^, and ^12^C^15^N^−^ using electron multipliers and a mass resolving power ~7,000 [1.5× correction, per (Pett-Ridge and Weber, 2012)]. Bacillus spores with known isotopic composition (previously analyzed by isotope-ratio mass spectrometry) were used as standards (Kreuzer-Martin and Jarman, 2007). Images were processed using L’Image software (developed by L Nittler, Carnegie Institution of Washington, Washington, DC, USA), including stitching together the individual 20 μm × 20 μm images to generate mosaics, identifying cells and cell boundaries using an automated particle-finder function in combination with hand-drawn regions of interest, and extracting isotope ratios.

### Determination of Activity and Metabolism Based on Isotopic Enrichment

Cellular isotopic ratios measured by nanoSIMS were converted to atom percent (at. %) and net assimilation percent (X_net_ %). At. % is the atomic fraction of the less abundant isotope in the measured biomass multiplied by 100, e.g., at. % ^15^N = [^15^N/(^15^N + ^14^N)] x 100. X_net_ % is a measure of the newly synthesized biomass relative to the final biomass for a given element X, e.g., N_net_ % = [*F*_s_/(*F*_s_ + *F*_i_)] × 100, where *F*_s_ is the fraction of N derived from the isotopically spiked substrate, and *Fi* is the fraction of N from the original biomass. *F*_s_ and *F*_i_ are defined mathematically in (Popa et al., 2007), and are dependent on the final and initial isotopic ratios of the cell, as well as the isotopic ratio of the isotopically spiked substrate. In practice, since isotopic analysis is destructive, an average isotope ratio for a representative sampling of cells at the initial time point is used instead of the initial isotopic composition of the exact cell. X_net_ % is similar to but distinct from Fx_net_ %, a term defined in Popa et al., 2007 to quantify newly synthesized biomass relative to the *initial* biomass: Fx_net_ % = *F*_s_/*F*_i_ × 100. X_net_ % can be readily calculated from Fx_net_ %: X_net_ % = Fx_net_ %/(Fx_net_ % + 100). Note, although the correct equation for Fx_net_ % was used in the analysis of (Popa et al., 2007), Equation 6 in that paper erroneously omits a “1–” in the last term of the denominator (Weber, personal communication). That term is included in the calculations here.

A cell was considered isotopically enriched, and therefore anabolically active, if the isotopic composition exceeded three times the standard deviation of the mean isotopic composition of unincubated/unlabeled cells from that site (*n* = 262 at SPOT; *n* = 216 at Pacifica). Net assimilation detection limits were determined based on the isotopic variability of the unincubated/unlabeled cells and the level of isotopic enrichment of the isotopically spiked substrate. The net assimilation detection limit at SPOT for C-derived from bicarbonate was 0.38%, and for N-derived from amino acids was 0.07%. The net assimilation detection limit at Pacifica for C-derived from bicarbonate was 0.69%, for N-derived from amino acids was 0.05%, and for C-derived from amino acids was 0.05%. As an example, an autotrophic cell at SPOT deriving all of its carbon from bicarbonate would have had a detectable isotopic enrichment if it synthesized just 0.38% of its biomass during the experiment.

Active cells were divided into four metabolic categories depending on their net assimilation of carbon versus nitrogen (C_net_ % and N_net_ % values) after incubation with ^13^C-bicarbonate and ^15^N-amino acids: (1) exclusively heterotrophic cell: C_net_ % < detection limit and N_net_ % > detection limit, i.e., 0% carbon derived from bicarbonate; (2) exclusively autotrophic cell: C_net_ % ≥ N_net_ %, i.e., 100% carbon derived from bicarbonate; (3) primarily heterotrophic cell: N_net_ % > C_net_ % × 2, i.e., <50% carbon derived from bicarbonate; (4) primarily autotrophic cell: C_net_ % < N_net_ % < C_net_ % × 2, i.e., >50% carbon derived from bicarbonate. Additional explanation for the definition of these categories is provided in the discussion.

## Results

### Cell Density

Initial cell density at SPOT was 5.80 ×10^5^ cells/ml (±1.01 ×10^5^). After 2 days of incubation with bicarbonate and amino acids, it was approximately half the initial value in both replicate bottles. After 7 days, it was essentially the same as the initial density in both replicates (Supplementary Table S4). For incubations treated with bacterial antibiotics, the cell density was not significantly lower at 7 days than in incubations without the treatment (Supplementary Table S4).

Initial cell density was not measured at Pacifica. After 3 days incubation with no amendments, the density was 3.84 ×10^6^ cells/ml (±0.72 ×10^6^), which was not statistically different from the Pacifica 3-day incubation amended with bicarbonate and amino acids, and was an order of magnitude higher than the SPOT samples (Supplementary Table S5). Cell densities were about 50% lower at 6 days than at 3 days at Pacifica. Similar to SPOT, antibiotics did not significantly change the cell density in Pacifica water incubated for 6 days (Supplementary Table S5).

### Initial Microbial Community Composition (DNA) and Potential Activity (RNA)

**Figure 1:**
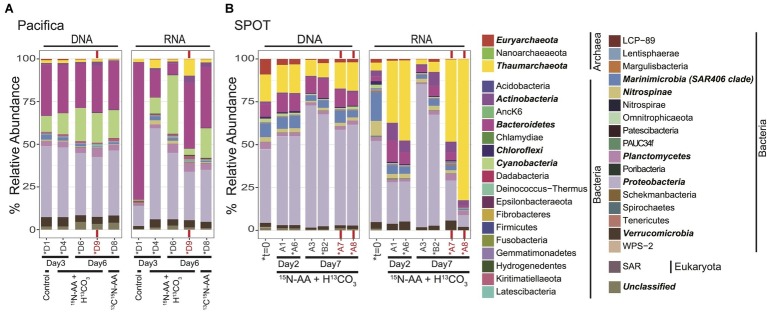
Total microbial community composition of seawater at t=0 and during isotope incubations, as assessed by 16S rRNA sequencing; **(A)** Pacifica and **(B)** SPOT. Each bar represents a single sample or biological replicate (technical replicates were averaged). Red text and bars indicate incubations amended with bacterial antibiotics. Controls in the Pacifica incubations were unamended. Asterisks indicate samples that were also analyzed *via* nanoSIMS. Phyla with a cumulative abundance >1% are indicated in bold. Total community includes Archaea, Bacteria, Eukaryota, and unclassified OTUs.

The initial (*t* = 0) microbial community at SPOT was assessed to determine the community composition prior to incubation. Proteobacteria was the most abundant bacterial phylum (43% of reads), followed by Marinimicrobia (SAR406 clade; 8% of reads), and Bacteroidetes (7% of reads) (Figure 1). The Proteobacteria were comprised primarily of Alphaproteobacteria [25% of reads, including SAR11 (21%)], Deltaproteobacteria [8% of reads, including SAR324, Marine Group B (4% of reads)], and Gammaproteobacteria [10% of reads, including Thiomicrospirales (3% of reads)] (data not shown). Although most of the bacterial lineages detected are likely heterotrophic, several autotrophic groups were also detected, including photoautotrophic Cyanobacteria (2% of reads) and chemoautotrophic Nitrospinae (3% of reads).

**Figure 2:**
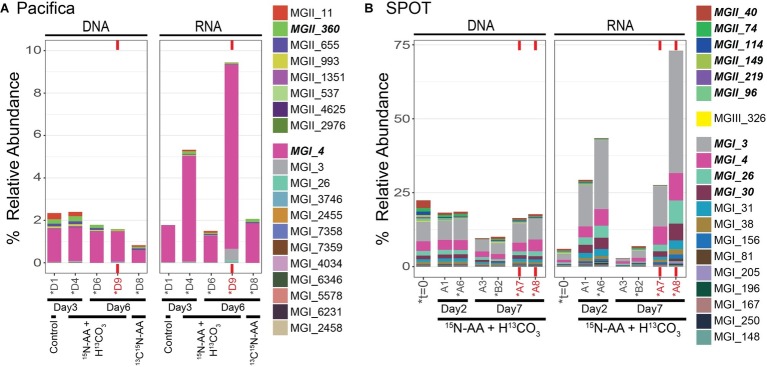
Top 20 most abundant archaeal OTUs at t=0 and during isotope incubations as assessed by 16S rRNA sequencing; **(A)** Pacifica and **(B)** SPOT. Each bar represents a single sample or biological replicate (technical replicates were averaged). Red text and bars indicate incubations amended with bacterial antibiotics. Relative abundance out of total microbial community. Colors are shared between sites. Asterisks indicate samples that were also analyzed *via* nanoSIMS. MGI OTUs with cumulative relative abundance >1% and MGII and MGIII OTUs >0.1% are indicated in bold in the legend.

Archaea comprised 25% of the total 16S rRNA gene reads detected at *t* = 0 at SPOT. The archaea were dominated by Thaumarchaeota MGI (16% of reads) and Euryarchaeota MGII and MGIII (9% of reads) (Figure 1). All 20 of the top most abundant archaeal operational taxonomic units (OTUs) belonged to these two phyla, with most belonging to the Thaumarchaeota (13/20) (Figure 2). The 20 most abundant OTUs comprised 91% of the total archaeal community, indicating a diversity of low abundance taxa within these phyla in the remaining 9%. The Thaumarchaeal OTUs at SPOT spanned both the previously described Shallow and Deep MGI phylogenetic clades (Swan et al., 2014), with the most abundant Thaumarchaeal OTU (MGI_3) clustering within the Deep clade (Supplementary Figure S2).

We used 16S rRNA as a proxy for potential activity. While 16S rRNA is thought to be a poor proxy for overall microbial activity, it can reflect protein synthesis potential (Blazewicz et al., 2013), which we call “potential activity” here. The most abundant groups detected in the DNA survey were again detected as the dominant groups in the RNA survey; however, their relative proportions were different. In particular, archaeal 16S rRNA reads comprised only 7% of the total detected. Within the archaea, the community composition of the top 20 OTUs was nearly identical between the DNA and RNA surveys (Supplementary Figure S3).

Samples were not preserved immediately after sampling at Pacifica Pier, and thus we do not have a measurement of the initial community composition. However, Pacifica seawater incubated with no chemical amendments was filtered and preserved for sequencing after 3 days. As in the initial SPOT sample, this community was dominated by Proteobacteria (41% of reads), and also contained Bacteroidetes (30% of reads), Cyanobacteria (9% of reads), Verrucomicrobia (5% of reads), Planctomycetes (3% of reads), and Marinimicrobia (SAR406 Clade; 3% of reads; Figure 1). Several differences between the SPOT *t* = 0 and Pacifica *t* = 3 day communities are notable, including the smaller percentage of total archaea (2.3% of total reads at Pacifica; 1.5% Thaumarchaeota, 0.8% Euryarchaeota) and the less diverse Thaumarchaeal population (a single OTU comprised 99% of Thaumarchaeotal reads) in the Pacifica sample (Figure 2, Supplementary Figure S3). This OTU (MGI_4) clustered within the previously described Shallow MGI phylogenetic clade (Supplementary Figure S2).

### Microbial Community Shifts During Incubation

We tracked changes in microbial community composition (16S rRNA genes) and potential activity (16S rRNA) during our incubations to assess shifts over time and between bottles with different chemical amendments. Within both SPOT and Pacifica seawater, the 16S rRNA gene profiles were remarkably stable at both the phylum (Figure 1) and OTU (Supplementary Figure S4) level throughout the week-long incubation, and despite the addition of isotopically labeled substrates. In particular, the composition of the archaeal community remained largely constant, maintaining the majority of its initial diversity, despite a decrease in relative abundance compared to bacteria (Figure 2; Supplementary Figure S3). The community 16S rRNA profile was substantially more dynamic than the community composition (Figures 1, 2, Supplementary Figure S4). Still, the 16S rRNA profile of archaeal OTUs was stable over time and despite addition of labeled substrates at both sites (Figure 2; Supplementary Figure S3). Replicate bottles at SPOT were highly self-consistent (Figures 1, 2; Supplementary Figures S3, S4).

We added bacterial antibiotics (ampicillin and streptomycin) to select incubations to enrich for archaea. In SPOT seawater, replicate incubations amended with bacterial antibiotics had proportionally more archaeal 16S rRNA genes (DNA) and 16S rRNA (RNA) relative to parallel incubations without antibiotics (Figures 1, 2). The increase in archaeal DNA was nearly 2-fold (average 1.7-fold), and in RNA was up to 27-fold (average 12-fold). At Pacifica, the same trend was observed in RNA but not DNA. In the RNA analysis at Pacifica, archaeal 16S rRNA was 6-fold more abundant relative to bacteria. Interestingly, these increases were driven solely by MGI Thaumarchaeota; MGII and MGIII Euryarchaeota maintained a constant relative abundance or decreased with the addition of antibiotics (Supplementary Figure S5).

### High-Throughput Metabolic Screening of Unidentified Cells by NanoSIMS

**Figure 3:**
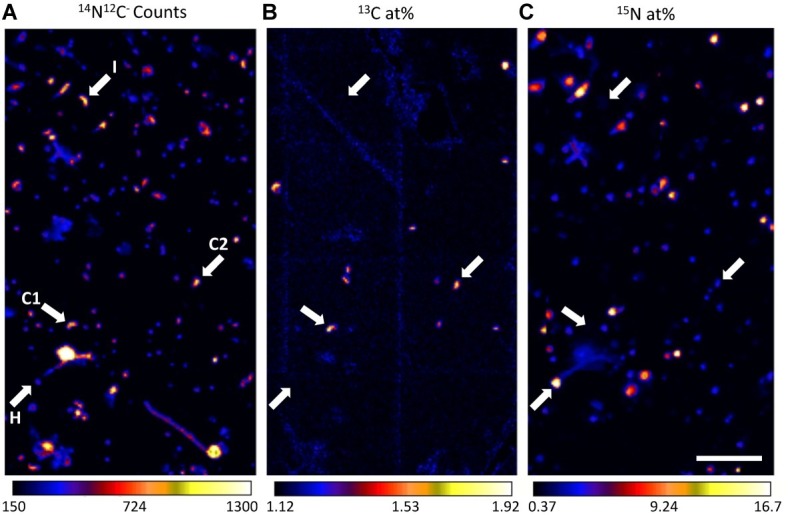
Representative nanoSIMS images demonstrating high-throughput metabolic screening of cells filtered from Pacifica seawater incubated with ^13^C-bicarbonate and ^15^N-amino acids for 6 days. Panel **(A)** shows ^14^N^12^C^−^ ion counts, reflecting all carbon- and nitrogen-containing particles, **(B)** ^13^C atom percent enrichment, indicates cells enriched in ^13^C, and **(C)** ^15^N atom percent enrichment, indicates cells enriched in ^15^N. The same four cells are indicated with arrows in each panel, with letters in panel **(A)** indicating their putative metabolism: I (no enrichment; inactive cell), C1 (enrichment in only ^13^C; chemoautotroph), H (enrichment in only ^15^N; heterotroph), and C2, (enrichment in ^13^C, minor enrichment in ^15^N; chemoautotroph). Scale bar is 11 µm.

**Figure 4:**
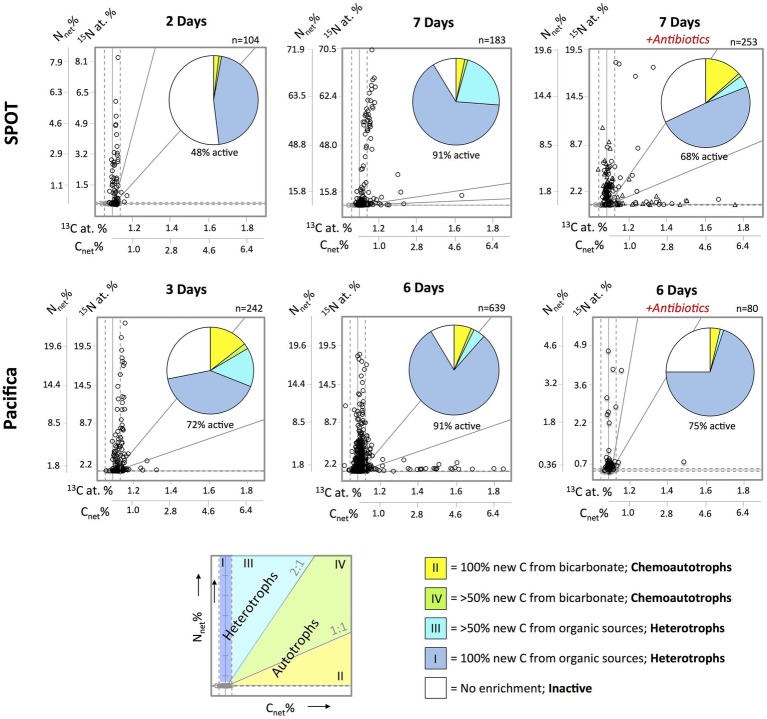
Metabolic characterization of individual cells (circles and triangles) filtered from SPOT and Pacifica seawater after incubation with ^15^N-amino acids and ^13^C-bicarbonate, based on net assimilation of C and N (C_net_% and N_net_%). Isotopically unlabeled cells from SPOT and Pacifica are plotted in gray; solid vertical and horizontal gray lines indicate average ^13^C at. % and ^15^N at. % for the unlabeled cells, dashed lines indicate 3 standard deviations from the mean in each direction. Biological replicates (separate incubations) are plotted in different shapes (circles and triangles) for SPOT. Pie charts show proportion of cells in each metabolic regime, according to the bottom schematic. Diagonal gray lines represent 1:1 and 2:1 N_net_%:C_net_%, as indicated in the schematic. The scale of the *y*-axis varies between plots in order to best demonstrate trends. Two cells were beyond the maximum ^13^C value in the SPOT 7 day plot with antibiotics and are not displayed.

**Figure 5:**
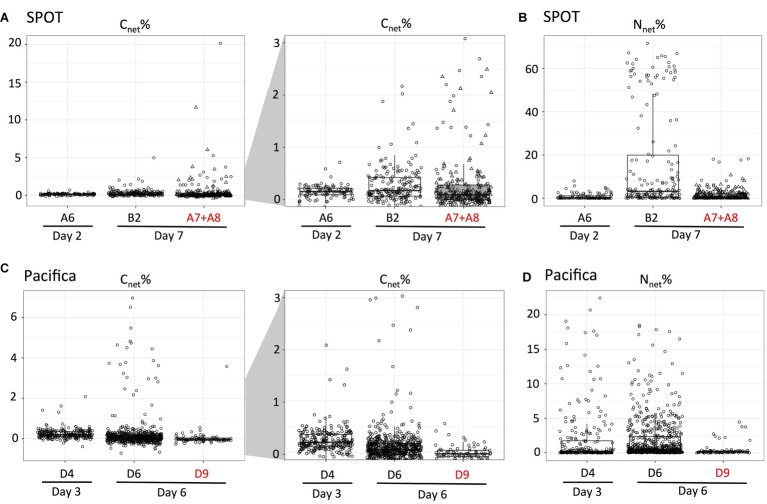
Box plots demonstrating the range of anabolic activity within and between experimental treatments. The C and N net assimilation of individual, unidentified fixed cells (circles and triangles) after incubation with ^13^C-bicarbonate and ^15^N-amino acids at SPOT **(A,B)** and Pacifica **(C,D)** is shown. ^13^C data are displayed twice with different y-axes. Incubations amended with bacterial antibiotics are indicated with red text. Two biological replicates are combined for the SPOT antibiotic treatment and differentiated with circles and triangles. Data are re-plotted from Figure 4.

We used nanoSIMS to analyze the carbon and nitrogen isotopic composition of 540 individual cells from SPOT and 961 individual cells from Pacifica incubated with ^13^C-bicarbonate and ^15^N-amino acids. Using the isotopic thresholds described here, we determined the fraction of the communities that was anabolically active, the range of activity across the cells, and the fraction of chemoautotrophs versus heterotrophs (Figures 3–5). Ninety-one percent of cells from both Pacifica and SPOT (after 6 and 7 days of incubation, respectively) demonstrated anabolic activity, i.e., incorporation of at least one isotopically labeled substrate (Figure 4). Heterotrophy was the dominant metabolism observed at both sites. Chemoautotrophy was also observed, comprising 6 and 5% of the active cells at SPOT at the 2- and 7-day time points, respectively, and 23 and 8% of the active cells at Pacifica at the 3- and 6-day time points, respectively (Figure 4).

The addition of bacterial antibiotics decreased the number of anabolically active cells from 91 to 75% at Pacifica, and from 91 to 68% at SPOT (Figures 4, 5). The metabolic profile of the community at SPOT also shifted in both replicates treated with antibiotics: the percent of the active community living chemoautotrophically (primarily ^13^C-enriched) increased to 25% in one incubation (bottle A7, *n* = 71 cells analyzed) and to 20% in the second (bottle A8; *n* = 182 cells analyzed), for a weighted average of 21% (Figure 4). We did not observe an increase in chemoautotrophy in the Pacifica incubation amended with antibiotics.

### Metabolic Characterization of Thaumarchaeal Cells by FISH-NanoSIMS

**Figure 6:**
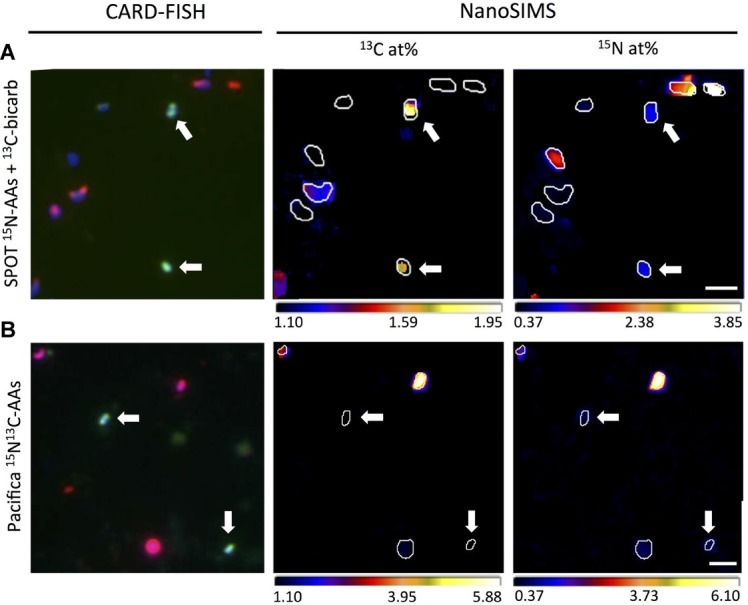
Representative correlated CARD-FISH and nanoSIMS images demonstrating isotopic composition of individual Thaumarchaeota cells (probe Cren547; green in CARD-FISH image; indicated by white arrows), bacterial cells (probe Eub338I; red in CARD-FISH image) and unidentified cells (DAPI; blue in CARD-FISH image). **(A)** Thaumarchaeota cells from SPOT incubated with ^13^C-bicarbonate and ^15^N-amino acids are enriched in both ^13^C and ^15^N, while most bacteria and unidentified cells are enriched only in ^15^N. **(B)** Most Thaumarchaeota cells from Pacifica Pier incubated with doubly labeled (^13^C and ^15^N) amino acids are enriched in ^15^N but not ^13^C, while bacteria and unidentified cells are enriched in both isotopes. Scale bar indicates 2 μm.

**Figure 7:**
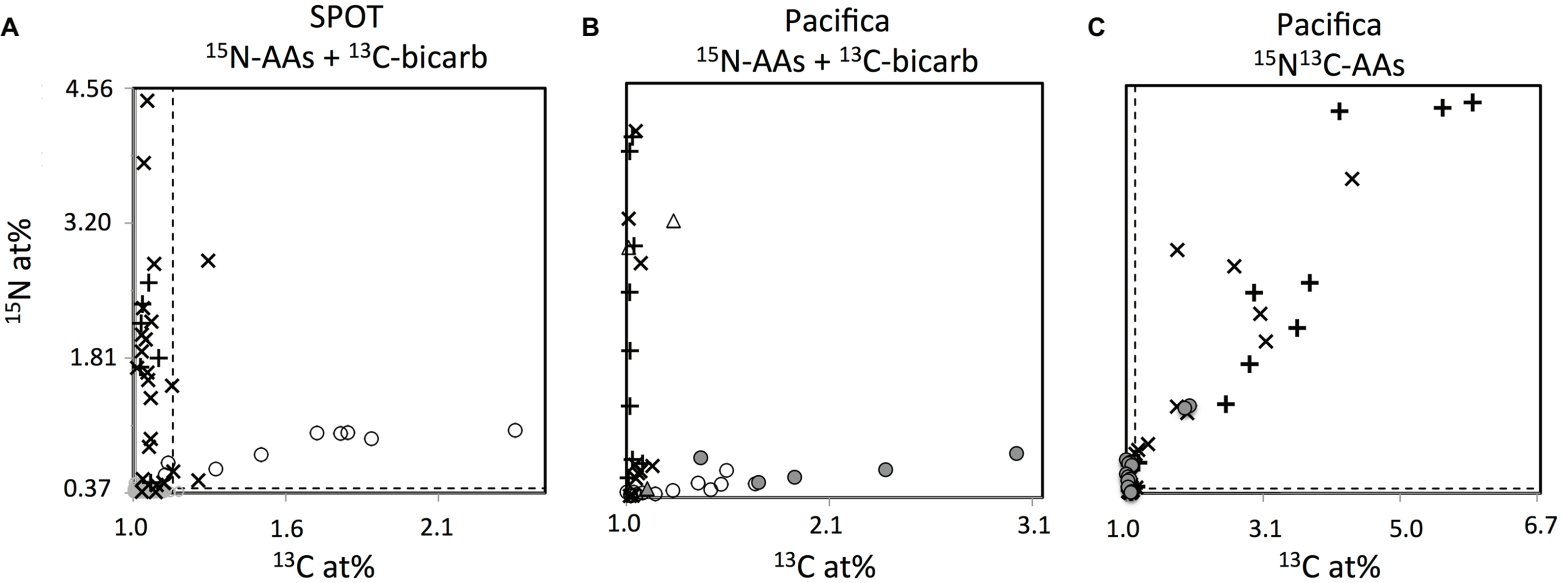
NanoSIMS measured isotopic composition expressed as atom percent (at. %) of individual cells identified as Thaumarchaeota (circles) or Bacteria (+’s), and unidentified DAPI-stained cells (×’s), from SPOT **(A)** and Pacifica **(B,C)**, after incubation with ^15^N-amino acids and ^13^C-bicarbonate **(A,B)** or ^13^C^15^N-amino acids **(C)**. Incubation times are as follows: **(A)** 2 days; **(B)** 3 days (all points except filled circles), 6 days (filled circles), and **(C)** 6 days. Three Thaumarchaeal cells that were co-located with other cells and whose isotopic composition may be derived from a combination of Thaumarchaeal and bacterial biomass are shown as triangles. Some bacterial cells with isotope values beyond the scale of the axes are not shown in **(C)** to best display the archaeal data. Dashed lines indicate thresholds for enrichment.

To specifically investigate metabolism in Thaumarchaeal cells, we analyzed the isotopic composition of 47 FISH-identified Thaumarchaeal cells, as well as 67 FISH-identified bacterial cells and 82 cells of unknown taxonomy (identified by DAPI staining but neither FISH probe; these cells may be members of the Archaea or Bacteria not targeted by the probes employed, or targeted organisms that failed to hybridize; Figures 6, 7). In SPOT seawater incubated with ^13^C-bicarbonate and ^15^N-amino acids, all FISH-nanoSIMS imaged Thaumarchaeota cells were anabolically active after 2 days (i.e., incorporated one or both isotopically-labeled substrates; 9/9 cells), and most were ^13^C enriched, suggesting chemoautotrophy (7/9 cells; Figure 7). In Pacifica seawater incubated with the same amendments, most but not all Thaumarchaeota cells were anabolically active after 3 days (12/16 cells); of these, just over half were ^13^C enriched (7/12 cells). However, after 3 additional days of incubation, all Thaumarchaeota cells investigated from Pacifica were anabolically active (6/6 cells), and almost all were enriched in ^13^C (5/6 cells).

Few cells other than FISH-identified Thaumarchaeota showed uptake of ^13^C-bicarbonate in water from either Pacifica or SPOT, suggesting that Thaumarchaeota were the primary chemoautotrophs at both sites. At SPOT, only 2 of 39 additional cells investigated were enriched in ^13^C, and neither hybridized with the bacterial probe, leaving open the possibility that they were actually unhybridized Thaumarchaeota. At Pacifica, none of the 63 additional cells we imaged were ^13^C-enriched after 3 or 6 days of incubation. At both sites, the percent of bacteria/unknown cells that were anabolically active was high (about 90% at Pacifica, 56/63 cells; and nearly 100% at SPOT, 38/39 cells).

To directly investigate the potential for carbon assimilation from amino acids by Thaumarchaea, we also analyzed FISH-identified Thaumarchaeota cells incubated with dual-labeled ^13^C-^15^N-amino acids. After incubation with ^13^C-bicarbonate and ^15^N-amino acids, all of the ^13^C-enriched Thaumarchaeota cells that were FISH-nanoSIMS imaged were also ^15^N-enriched (7/7 cells at SPOT, 13/13 cells at Pacifica). Therefore, as expected, when incubated with dual-labeled ^13^C-^15^N-amino acids, nearly all Thaumarchaeota were ^15^N-enriched (13/15 cells, Pacifica only). However, of these, nearly all failed to incorporate the ^13^C derived from the same molecule (11/13 Thaumarchaeota cells were ^15^N enriched but not ^13^C enriched). In contrast, most bacterial and unidentified cells that were enriched in ^15^N also took up ^13^C from the dual-labeled substrate in the same incubation (14/16 unidentified cells, 11/13 bacterial cells).

## Discussion

### High-Throughput Single-Cell Characterization of Metabolism Using Multi-isotope NanoSIP: Concept and Definition of Thresholds

Isotope tracer techniques are regarded as some of the most definitive assays for microbial activity in environmental samples. Depending on the substrate, tracking the assimilation of an isotopically labeled substrate can provide a measure of overall anabolic activity [e.g., ^15^NH_4_^+^ (Krueger et al., 2008; Orphan et al., 2009) and D_2_O (Berry et al., 2015; Kopf et al., 2015)] or a test of specific metabolic capabilities [e.g., ^15^N_2_ (Dekas et al., 2009, 2018), ^13^CH_4_ (Hutchens et al., 2004), H^13^CO_3_^−^ (Wuchter et al., 2003)]. Traditional isotope tracer techniques such as DNA-based stable isotope probing (DNA-SIP) and microautoradiography (MAR) cannot distinguish between multiple isotope labels at once, and therefore cannot differentiate between inactivity and inability. However, more recent SIP techniques, such as nanoSIMS imaging (nanoSIP) and lipid analysis (lipid-SIP), greatly benefit from the simultaneous use of several isotopically labeled substrates. “Multi-isotope SIP” allows for the differentiation of several specific metabolisms at once, and through thoughtful experimental design, one can identify and quantify otherwise elusive metabolisms and distinguish between nuanced phylotypes (e.g., Kopf et al., 2015; Dawson et al., 2016; Wegener et al., 2016). Despite the frequent application of multi-isotope nanoSIP in relatively low-throughput analyses targeting particular taxa (e.g., dozens of FISH-identified cells), it has rarely been used as a high-throughput metabolic screening technique to characterize hundreds to thousands of individual, unidentified cells (Dawson et al., 2016; Arandia-Gorostidi et al., 2017). Used in this manner, it has the potential to analyze enough cells to generate metabolic activity profiles representative of the entire community.

Here, we use high-throughput multi-isotope nanoSIP to rapidly characterize individual marine cells as active/inactive and chemoautotrophic/heterotrophic based on their relative enrichment in ^13^C and ^15^N after incubation with ^13^C-bicarbonate and ^15^N-amino acids. This approach builds on the concept of lipid “dual-SIP,” which quantifies the production of lipids as heterotrophic or autotrophic based on incorporation of ^13^C from ^13^C-bicarbonate, as an indicator of autotrophy, and/or deuterium from D_2_O, as a general indicator of activity (Kellermann et al., 2012; Wegener et al., 2012, 2016). Heterotrophy is measured as the difference between the active and autotrophic fractions. Not all heterotrophs assimilate the same organic substrates, so this approach is more inclusive than quantifying the uptake of specific ^13^C-labeled substrates. Applying this concept on the single-cell level (i.e., nanoSIP), rather than at the community or population level (i.e., lipid-SIP), can resolve the *variability* in community activity in addition to metabolic preferences. For instance, a bulk approach may over-estimate the ecological significance of heterotrophy in a community with few but highly productive heterotrophic cells despite numerical dominance of slow-growing, autotrophic cells.

A challenge of stable isotope probing experiments is the possibility of substrate recycling, i.e., the transfer of the isotope label from one molecule to another *via* enzymatic activity (also known as cross-feeding). Short incubation times are preferable to minimize this. However, incubation times must be long enough to observe anabolic activity, and ideal incubation length is therefore habitat- and even organism-specific. We conducted incubations from 2 to 7 days due to the expected slow growth of cold-adapted natural microbial communities, and specifically chemoautotrophs. Indeed, microbial turnover times at 100 m ocean depth have been estimated to be 17–99 days (Nagata et al., 2000; Reinthaler et al., 2006), and the turnover time at SPOT at the time of sampling was 38 days (Parada and Fuhrman, 2017). Still, since some taxa are more active than others, we assume that substrate recycling occurred during the experiments. Specifically, we assume that the added ^15^N-amino acids were converted to a variety of ^15^N-labeled molecules, including ^15^N-ammonium, through several potential mechanisms (e.g., Palenik and Morel, 1991; Mulholland et al., 1998, 2003). We therefore interpret ^15^N-enrichment after incubation with single-labeled ^15^N-amino acids in these experiments as a general indicator of anabolic activity, rather than the assimilation of an intact amino acid. However, we do interpret ^13^C enrichment in these incubations specifically as assimilation of bicarbonate. The ^13^C-enrichment in Thaumarchaeota in these incubations is unlikely due to recycling because no cells except the Thaumarchaeota are ^13^C-enriched, suggesting that they were the only active chemoautotrophs.

In order to rapidly characterize a cell’s metabolism based on its isotopic composition, we established quantitative thresholds for classification. For cells incubated with ^15^N-amino acids and ^13^C-bicarbonate, N_net_ % indicates the percent of new biomass generated, while C_net_ % indicates the percent synthesized autotrophically. When N_net_ % and C_net_ % are equal, it indicates that all of the carbon in the cell’s newly synthesized biomass was derived from bicarbonate, and it was therefore growing autotrophically. This assumes the newly synthesized biomass has the same C:N ratio as the existing biomass, but otherwise is independent of the C:N ratio of the cell.

We identified cells as chemoautotrophic if all (100%; i.e., C_net_ % ≥ N_net_ %; Zone II in Figure 4) or most (≥50%; i.e.; C_net_ % < N_net_ % < C_net_ % × 2; Zone IV in Figure 4) of the carbon in their newly synthesized biomass was derived from bicarbonate. If additional, unlabeled nitrogen sources were also assimilated (for instance, if the standing stock of amino acids or ammonium were not zero), N_net_ % may be underestimated, leading to C_net_ % greater than N_net_ %. However, in this case the cell would still be accurately classified as a chemoautotroph. Our classification of chemoautotrophs includes cells which assimilate up to 50% of their carbon from organic sources (Zone IV) to account for autotrophs who uptake some organic matter, like amino acids. However, very few cells fell within this metabolic regime in either sample.

In the same incubations—^13^C-bicarbonate and ^15^N-amino acids—we classified a cell as heterotroph if all (100%; i.e., N_net_ % > 0 and C_net_ % = 0; Zone I in Figure 4), or most (>50%; i.e., N_net_ % > C_net_ % × 2; Zone III in Figure 4) of the carbon in its newly synthesized biomass was derived from organic carbon. Anaplerotic reactions, i.e., replenishment of metabolic intermediates, can result in minor (~1–3%) inorganic carbon fixation by heterotrophs, and might explain the cells in the latter category (Krebs, 1941; Werkman and Wood, 2000; Hesselsoe et al., 2005). Mixotrophy could also explain cells in Zone III and Zone IV. However, the ^13^C-enrichment observed in heterotrophs here is extremely minimal compared to their ^15^N-enrichment, and therefore suggests anaplerotic reactions rather than mixotrophy. Overall, the close clustering of the cells along one or the other axis of enrichment in Figure 4 suggests that although some inorganic carbon is assimilated by heterotrophs and some organic carbon by autotrophs, this activity is minimal. Combined with the potential for some amount of substrate recycling, we do not specifically identify mixotrophs in this dataset, and rather classify cells by their primary carbon source: inorganic (chemoautotrophic) or organic (heterotrophic).

### Applying High-Throughput Multi-isotope NanoSIP to Characterize Microbial Community Activity and Metabolism in Pacific Ocean Water

Quantifying the number of active cells in a natural sample is challenging, and therefore this number is currently poorly constrained in most habitats. Previous studies have suggested that 20–30% of DAPI-stained marine cells are dead, based on the observation of damaged internal structures by transmission electron microscopy (Heissenberger et al., 1996) and a lack of hybridization during FISH (Herndl et al., 2005). However, we found that most individual cells in our samples (>90%) were anabolically active in our incubations (Figure 4). And, this value is likely to be an underestimate given the conservative threshold we used to identify isotopic enrichment (>3× the standard deviation of the mean of unlabeled cells). We even observed anabolic activity in cells that failed to hybridize during FISH (Figure 7), indicating specifically that FISH estimates of active cells may be underestimates. By necessity, these observations were conducted in bottle experiments, and are therefore likely to have deviations from *in situ* observations. However, these observations suggest that dormant cells in marine waters are rare. Additionally, we found activity levels between cells were highly variable, ranging several orders of magnitude (Figure 5). This indicates that although most cells may be active, a relatively small percentage of cells are responsible for a disproportionately large fraction of the total anabolic activity.

Chemoautotrophic cells comprised 7–17% of the community in Pacifica water, and 3–4% of that in SPOT water. These values were higher than expected at Pacifica, where <5% of the community 16S rRNA gene reads were comprised by known chemoautotrophs, and lower than expected at SPOT, where putatively chemoautotrophic Thaumarchaeota comprised 10–15% of the 16S rRNA gene reads. A number of factors could explain the discrepancies. Chemoautotrophy may have been overestimated by nanoSIMS if some photoautotrophic activity contributed to bicarbonate assimilation. Incubations were kept in the dark once the isotopically labeled substrates were added, but not acclimatized to darkness before. This leaves open the possibility that reducing equivalents were initially still available for carbon fixation by photoautotrophs. Conversely, chemoautotrophy may be underestimated, since the nanoSIMS analysis was more sensitive to ^15^N-enrichment from amino acids (i.e., activity) than ^13^C-enrichment from bicarbonate (i.e., autotrophy), due to the higher percentage of isotopic labeling of the former. Autotrophs with low levels of activity therefore could have been identified as active but not autotrophic. Additionally, discrepancies between the sequencing and nanoSIMS datasets could be due to inaccuracies in sequencing relative abundances due to primer bias, or incorrect assumptions about the metabolism of the sequenced microbes.

We manipulated the community composition by amending some incubations with bacterial antibiotics in order to observe parallel changes in community composition (observed by sequencing) and the metabolic profile (observed by nanoSIMS). In particular, we hypothesized that if most Thaumarchaeota were chemoautotrophic, increasing the relative abundance of Thaumarchaea in the sample would cause an increase in the relative number of chemoautotrophs detected. The antibiotics ampicillin and streptomycin inhibit bacterial cell wall synthesis and bacterial protein synthesis, respectively, with little to no effect on archaea (or eukaryotes). These antibiotics have been used to establish and maintain enrichments of Thaumarchaeota from pelagic marine samples (Santoro and Casciotti, 2011; Berg et al., 2014; Ahlgren et al., 2017). We used a single dose of ampicillin and streptomycin in select bottles at the beginning of our incubation experiment and tracked changes in the community composition (16S rRNA sequence profile) and activity (assimilation of isotopically labeled substrates) over time.

As expected, the relative abundance (16S rRNA gene) and relative potential activity (16S rRNA) of archaea increased with the addition of antibiotics in SPOT water, as did the relative potential activity of archaea in Pacifica water (Figures 1, 2). In duplicate incubations of SPOT water, the average 2-fold increase in MGI relative abundance and average 15-fold increase in MGI relative potential activity with the addition of antibiotics was matched by a 3.5-fold increase in the percentage of chemoautotrophs detected by nanoSIMS. Since no other taxa increased as significantly in relative abundance, this is strong circumstantial evidence that members of the MGI Thaumarchaeota at SPOT were chemoautotrophic. In Pacifica water, an increase in chemoautotrophy with the addition of antibiotics was not observed by nanoSIMS, but was not expected there since an increase in MGI relative abundance was not observed. Although these correlations are not definitive, they demonstrate the utility of this technique to observe overall trends and generate testable hypotheses. These can be pursued with more laborious and definitive measures to link taxa and activity, such as FISH-nanoSIMS, as we describe in the next section.

### Carbon Metabolism of the Thaumarchaeota Assessed by FISH-NanoSIMS

FISH-nanoSIMS directly confirmed the assimilation of bicarbonate by most active Thaumarchaeota (80%) in water from both Pacifica and SPOT. Chemoautotrophy has been previously demonstrated in marine Thaumarchaeota, including by growth of pure cultures on inorganic carbon coupled to ammonia oxidation (Konneke et al., 2005; Santoro and Casciotti, 2011; Mosier et al., 2012; Berg et al., 2014) and bulk isotopic composition of both natural and ^13^C-labeled archaeal populations (Pearson et al., 2001; Wuchter et al., 2003; Ingalls et al., 2006). Genomic and metagenomic analyses indicate that Thaumarchaeota fix inorganic carbon *via* a modified 3-hydroxypropionate, 4-hydroxybutyrate pathway (Hallam et al., 2006; Blainey et al., 2011; Konneke et al., 2014; Swan et al., 2014; Li et al., 2015). The lack of bicarbonate assimilation in the remaining 20% of active Thaumarchaeal cells analyzed here could be due to the presence of some non-chemoautotrophic Thaumarchaeota, or more likely, varying detection limits of activity and autotrophy in this assay, as described above.

However, several lines of previous evidence suggest some level of organic matter assimilation by Thaumarchaeota, either *via* universal mixotrophy or by mixotrophy or heterotrophy in a subgroup of the phylum (Ouverney and Fuhrman, 2000; Teira et al., 2004; Herndl et al., 2005; Hallam et al., 2006; Ingalls et al., 2006; Kirchman et al., 2007; Alonso-Saez et al., 2008; Swan et al., 2014; Li et al., 2015; Seyler et al., 2018). To test whether Thaumarchaeota were capable of assimilating amino acids in our samples, we performed experiments with dual-labeled amino acids (i.e., both ^13^C and ^15^N). ^15^N enrichment indicated if the cell was anabolically active, as described above, while ^13^C enrichment indicated amino acid assimilation. Biomass enriched in ^13^C indicated the direct consumption of amino acids because even 100% recycling of the added ^13^C-amino acids to ^13^C-bicarbonate by heterotrophic bacteria would not have been enough to sufficiently change the isotopic composition of the large natural pool of bicarbonate (approximately 2.3 mM) to result in ^13^C enrichment by autotrophy.

Bacteria were enriched in both ^13^C and ^15^N after incubation with the dual-labeled amino acids, consistent with previous studies (e.g., Mayali et al., 2013). But, in the same incubations, only 15% of the Thaumarchaeal cells enriched in ^15^N were also enriched in ^13^C. This demonstrates that although they were active, most of the Thaumarchaeota cells did not assimilate carbon from amino acids. The ^15^N assimilation in the Thaumarchaeota cells may have been due to assimilation of ^15^NH_4_^+^ generated by heterotrophic bacteria (i.e., recycling), or potentially by intracellular or extracellular cleavage of amino acids by the Thaumarchaeota themselves. Regarding the latter, a growing body of evidence suggests that natural populations of MGI Thaumarchaeota use urea as a substrate for nitrification, producing ammonia for oxidation *via* urease (Alonso-Saez et al., 2012; Tolar et al., 2017). It is therefore feasible that they also use other forms of organic nitrogen, such as amino acids, for this purpose, and that some amino acid-derived ammonia would be assimilated in the process. Although an intriguing possibility, our dataset cannot differentiate between Thaumarchaeota-specific cleavage of amino acids for nitrification, and more general substrate recycling within the community.

The Thaumarchaeal population investigated for ^13^C^15^N-amino acid assimilation was from Pacifica, where a single OTU, MGI_4, comprised the vast majority of the Thaumarchaeal reads (99%). Therefore, the fact that most active Thaumarchaeal cells (85%) did not assimilate amino acid-derived carbon suggests specifically that most MGI_4 cells cannot assimilate this carbon, or at least did not under the conditions of the incubation. The uptake in the remaining 15% of active Thaumarchaeota suggests physiological heterogeneity within MGI_4, or within the greater phylum, if the nanoSIMS analysis included other Thaumarchaeal phylotypes potentially underestimated in relative abundance by the amplicon sequencing analysis. The former would indicate metabolic heterogeneity within the Thaumarchaoeta at a fine phylogenetic resolution. Another possible interpretation is that assimilation of amino acids is dependent on growth stage. The 15% of Thaumarchaeota cells that were enriched in ^13^C—implying uptake of amino-acid-derived carbon—were more enriched in ^15^N than the other Thaumarchaeota cells examined, and therefore more anabolically active (Figure 7).

Our observation that most active Thaumarchaeota did not incorporate carbon from amino acids appears to be in contrast to previous marine studies that have observed amino acid assimilation (Ouverney and Fuhrman, 2000; Teira et al., 2004; Herndl et al., 2005; Kirchman et al., 2007). The phylogenetic composition of Thaumarchaeota varies across marine samples – particularly with water depth (Beman et al., 2008; Swan et al., 2014; Parada and Fuhrman, 2017) – and different studies assessing Thaumarchaeota in different samples could therefore have discrepancies due to intra-phylum physiological heterogeneity. But in fact, the previous studies also reported amino acid uptake in only a sub-group of the Thaumarchaeal cells investigated—60% at 200 m water depth (Ouverney and Fuhrman, 2000), 3–18% from 200 to 3,000 m water depth (Herndl et al., 2005), and ~10% from 100 to 4,000 m water depth (Teira et al., 2004). The results are therefore consistent, and the difference lies in the interpretation. Previously, the observation of amino acid uptake in less than all cells could be attributed to the presence of inactive Thaumarchaeota. These previous studies used a single isotope tracer (tritiated-amino acids), and therefore were not able to independently assess activity. Our multi-isotope analysis demonstrates lack of amino acid incorporation even within active Thaumarchaeota. Therefore, the most likely interpretation of our results as well as that of the previous studies is intra-phylum heterogeneity in amino acid utilization.

## Conclusion

Using a high-throughput multi-isotope single-cell metabolic screening technique, we generated microbial anabolism profiles for environmental samples under near *in situ* and experimentally manipulated conditions. This technique is a valuable application of the nanoSIP approach, and can be widely applied. It would be particularly useful in environments with high percentages of uncharacterized lineages, where 16S rRNA sequences are unusually poor predictors of metabolism, and/or where microbial activity is low or variable. It could also be valuable in systems where active community functional genes/transcripts are conflated with those of a dormant community. In combination with DNA sequence data and/or experimental manipulations, this technique can generate hypotheses regarding which phylogenetic groups are responsible for specific metabolisms, testable by targeted techniques such as FISH-nanoSIMS.

Using this combined approach, we determined that the vast majority of cells in samples from both the coastal marine euphotic and aphotic zones are anabolically active during dark bottle incubations, and quantified the number of chemoautotrophic and heterotrophic cells. We found that nearly all of the active Thaumarchaeota were chemoautotrophic, even within the highly diverse Thaumarchaeota population within the aphotic sample. We additionally observed uptake of ^15^N but not ^13^C in most Thaumarchaeotal cells when incubated with ^13^C^15^N-amino acids in the euphotic sample, in contrast to the dual uptake observed in bacterial cells. This shows that not all active marine Thaumarchaeota assimilate intact amino acids. It also raises questions about the use of amino acids for catabolic purposes by Thaumarchaeota. Additional experiments using ^13^C-organic matter, which is not as susceptible to recycling effects in the marine environment as that labeled with ^15^N, will be useful to further characterize if and how diverse environmental populations of Thaumarchaeota utilize organic substrates.

## Data Availability Statement

The datasets generated for this study can be found in European Molecular Biology Laboratory, PRJEB31502.

## Author Contributions

AD conceived of the study, collected the samples, performed the experiments, processed samples, analyzed data, and wrote the manuscript. AP and JW processed samples. AP also analyzed data and contributed to figure generation. JF provided the sampling opportunity and contributed to the experimental design and data interpretation. XM, PW, and JP-R contributed to experimental design, nanoSIMS analysis, and data interpretation. All authors edited the manuscript.

### Conflict of Interest

The authors declare that the research was conducted in the absence of any commercial or financial relationships that could be construed as a potential conflict of interest.
