# Supplementary material for: Characterizing Chemoautotrophy and Heterotrophy in Marine Archaea and Bacteria With Single-Cell Multi-isotope NanoSIP
Source: Front Microbiol. 2019 Dec 17;10:2682. doi: 10.3389/fmicb.2019.02682 (PMC6927911; doi:10.3389/fmicb.2019.02682)

Characterizing chemoautotrophy and heterotrophy in marine archaea and bacteria with single-cell multi-isotope nanoSIP

Anne E. Dekas[1,2], Alma Parada[1,3], Xavier Mayali[2], Jed A. Fuhrman[3], Jessica Wollard[2], Peter Weber[2], Jennifer Pett-Ridge[2]

[1]*Department of Earth System Science, Stanford University, Stanford, CA 94305*
[2]*Physical and Life Sciences Directorate, Lawrence Livermore National Laboratory, Livermore, CA 94550*
[3]*Department of Biological Sciences, University of Southern California, Los Angeles, CA 90089*

Supplementary Figures and Tables

Tables

Table S1. SPOT Contextual Data. BDL indicates below detection limit. Detection limit for ammonium was 10 nM.

Table S2. Summary of experiments and analyses.

Table S3. Average Bray-Curtis Similarities between replicate mock communities and samples. The number of pairwise comparisons are given by n. All replicates include PCR replicates, different filter extractions of the same sample, and different elutions of the same extraction.

Table S4. Cell density over time in bottle incubations with SPOT seawater. Results for duplicate incubations are shown.

Table S5. Cell density over time in bottle incubations with Pacifica seawater.

Figures

Figure S1. Sampling sites. (A) Map of the western United States indicating locations of Pacifica Pier (Pacifica) and the San Pedro Ocean Time Series (SPOT). (B) Pacifica Pier. (C) Niskin rosette at SPOT.

Figure S2. Maximum-likelihood 16S rRNA gene phylogenetic tree of the most abundant Thaumarchaeal (MGI) OTUs detected in either Pacifica or SPOT. Sequences from this study are shown in red. Shallow and Deep marine groups designated as in Swan et al (2014). Reference sequences are italicized and NCBI accession numbers are included in tip labels. Bootstrap values >50% are shown (1000 replicates).

Figure S3. Top 20 most abundant archaeal OTUs *in situ* and during incubation as assessed by 16S rRNA sequencing of seawater from (A) Pacifica and (B) SPOT. Data is rescaled from Figure 2 to better demonstrate the stability of the archaeal community composition. Each bar represents a single sample or biological replicate (technical replicates were averaged). Red text and bars

indicate incubations amended with bacterial antibiotics. Relative abundance out of total community. Colors are shared between sites. Asterisks indicate samples that were also analyzed via nanoSIMS.

Figure S4. OTU-level changes in community composition over time and between treatments. Relative abundance of the top 50 most abundant OTUs and NMDS ordination of the Bray-Curtis Dissimilarity of the microbial communities *in situ* and during incubation as assessed by 16S rRNA sequencing of seawater from (A,B) Pacifica and (C,D) SPOT are shown. Bray-Curtis Dissimilarity was calculated using all OTUs in a sample. The number adjacent to each symbol in the NMDS plots indicates the day that sample was taken.

Figure S5. Archaeal community composition *in situ* and during incubation as assessed by 16S rRNA sequencing separated by the three major archaeal groups detected: MGI Thaumarchaeota (A+D), MGII Euryarchaeota (B+E) and MGIII Euryarchaeota (C+F), at both Pacifica (A-C) and SPOT (D-F). Shown as relative abundance of archaeal OTUs. The top 20 most abundant OTUs are shown for MGI and MGII, and the top 10 for MGIII. Each color represents a unique OTU.

Table S1. SPOT Contextual Data. BDL indicates below detection limit. Detection limit for ammonium was 10 nM.

Measurement Description	Value	Units	Reference	Method
Depth of chlorophyll fluorescence max	45	m	Parada et al 2017	CTD measurement
Ammonium concentration	BDL	μM	This Study	Standard fluorometric method, Holmes et al. (1999)
Nitrite concentration	BDL	μM	Parada et al 2017	Standard colorimetric measurement, Parsons et al. (1984)
Nitrate concentration	18.485	μM	Parada et al 2017	Standard colorimetric measurement, Parsons et al. (1984)
Oxygen concentration	3.1142	ml/l	Parada et al 2017	CTD measurement
Phosphate concentration	1.48	mM	Parada et al 2017	Standard colorimetric measurement, Parsons et al. (1984)
Salinity	33.7329	PSU	Parada et al 2017	CTD measurement
Temperature	9.4716	°C	Parada et al 2017	CTD measurement
Bacterial productivity	9779.43	cells/ml/day	Parada et al 2017	Leucine incorporation, Fuhrman et al. (2006)
Cell turnover	38.1	Day	Parada et al 2017	Cell abundance divided by leucine incorporation, Fuhrman et al. (2006)

Table S2. Summary of experiments and analyses.

			Incubation Amendments					Analyses			
	Name	Incubation Time (Days)	^{13}C-bicarbonate (% final)	^{15}N-Amino Acids (nM added)	^{13}C-^{15}N-Amino Acids (nM added)	Streptomycin (mg/ml)	Ampicilin (mg/ml)	Cell enumeration	16S rRNA survey	High-Throughput NanoSIMS Metabolic Screening	FISH-NanoSIMS
SPOT	--	0						Y	Y	Y	
	A6	2	13	50				Y	Y	Y	Y
	A1	2	13	50				Y	Y		
	B2	7	13	50				Y	Y	Y	
	A3	7	13	50				Y	Y		
	A7	7	13	50		0.05	0.05	Y	Y		
	A8	7	13	50		0.05	0.05	Y	Y	Y	
Pacifica	D1	3						Y	Y	Y	
	D4	3	7.5	100				Y	Y		Y
	D6	6	7.5	100				Y	Y	Y	Y
	D9	6	7.5	100		0.05	0.05	Y	Y		
	D8	6			104			Y	Y	Y	Y

Table S3. Average Bray-Curtis Similarities between replicate mock communities and samples.The number of pairwise comparisons are given by n. All replicates include PCR replicates, different filter extractions of the same sample, and different elutions of the same extraction.

	Bray Curtis Similarity<br>Mean $\pm$ SE (n)
Even Mock Communities	90.9 $\pm$ 0.260 (190)
Staggered Mock Communities	90.4 $\pm$ 0.245 (210)
Pacifica and SPOT<br>PCR Replicates	74.1 $\pm$ 4.9 (6)
Pacifica and SPOT<br>All Replicates	73.6 $\pm$ 0.85 (30)

Table S4. Cell density over time in bottle incubations with SPOT water. Results for duplicate incubations are shown.

	Chemical Amendments									
	Unamended		^{13}C-bicarbonate + ^{15}N-Amino Acids				^{13}C-bicarbonate + ^{15}N-Amino Acids + Antibiotics			
Incubation Time (days)	cells/ml x10^{5}	SD x10^{5}	cells/ml x10^{5}	SD x10^{5}	cells/ml x10^{5}	SD x10^{5}	cells/ml x10^{5}	SD x10^{5}	cells/ml x10^{5}	SD x10^{5}
0	5.80	1.01								
2			2.98	0.43	2.62	0.30				
7			4.79	0.53	5.29	0.64	4.81	0.72	3.74	0.52

Table S5. Cell density over time in bottle incubations with Pacifica water.

	Chemical Amendments									
	Unamended		^{13}C-bicarb + ^{15}N-AAs		^{13}C-bicarb + ^{15}N-AAs + Antibiotics		^{13}C-^{15}N-AAs		^{13}C-^{15}N-AAs + Antibiotics	
Incubation Time (days)	cells/ml x10^{6}	SD x10^{6}	cells/ml x10^{6}	SD x10^{6}	cells/ml x10^{6}	SD x10^{6}	cells/ml x10^{6}	SD x10^{6}	cells/ml x10^{6}	SD x10^{6}
0	3.84	0.72								
3	5.57	1.41	4.58	1.69	2.44	0.43				
6			2.44	0.64	1.12	0.29	2.37	0.24	1.75	0.48

Figure S1. Sampling sites. (A) Map of the western United States indicating locations of Pacifica Pier (PP) and the San Pedro Ocean Time Series (SPOT). (B) Pacifica Pier. (C) Niskin rosette at SPOT.

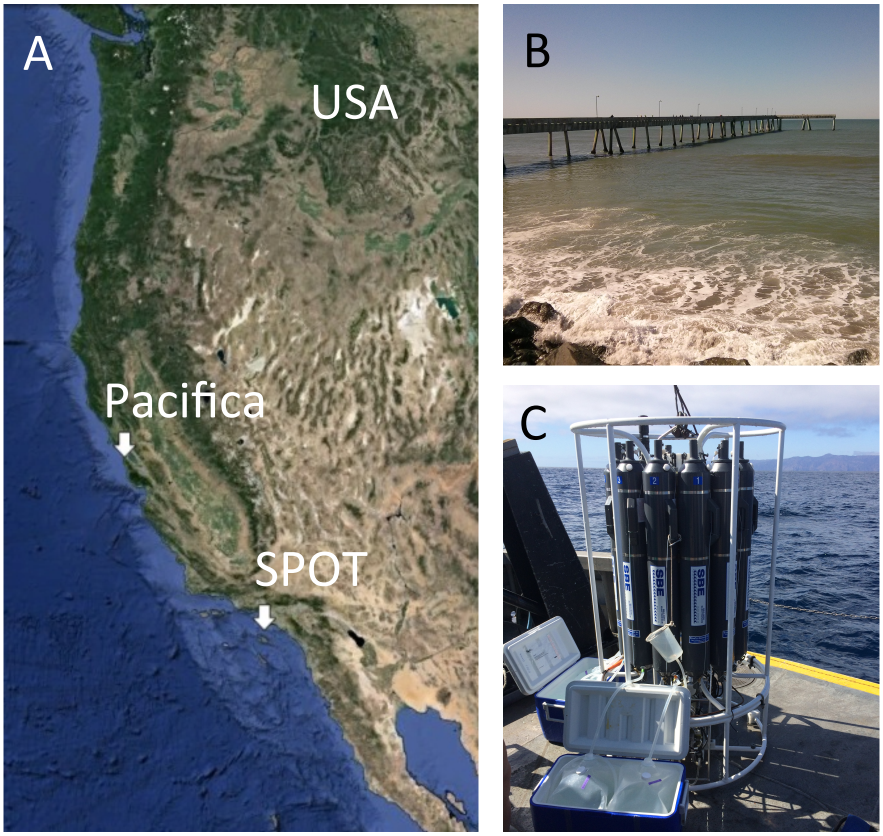

Figure S2. Maximum-likelihood 16S rRNA gene phylogenetic tree of the most abundant Thaumarchaeal (MGI) OTUs detected in either Pacifica or SPOT. Sequences from this study are shown in red. Shallow and Deep marine groups designated as in Swan et al (2014). Reference sequences are italicized and NCBI accession numbers are included in tip labels. Bootstrap values >50% are shown (1000 replicates).

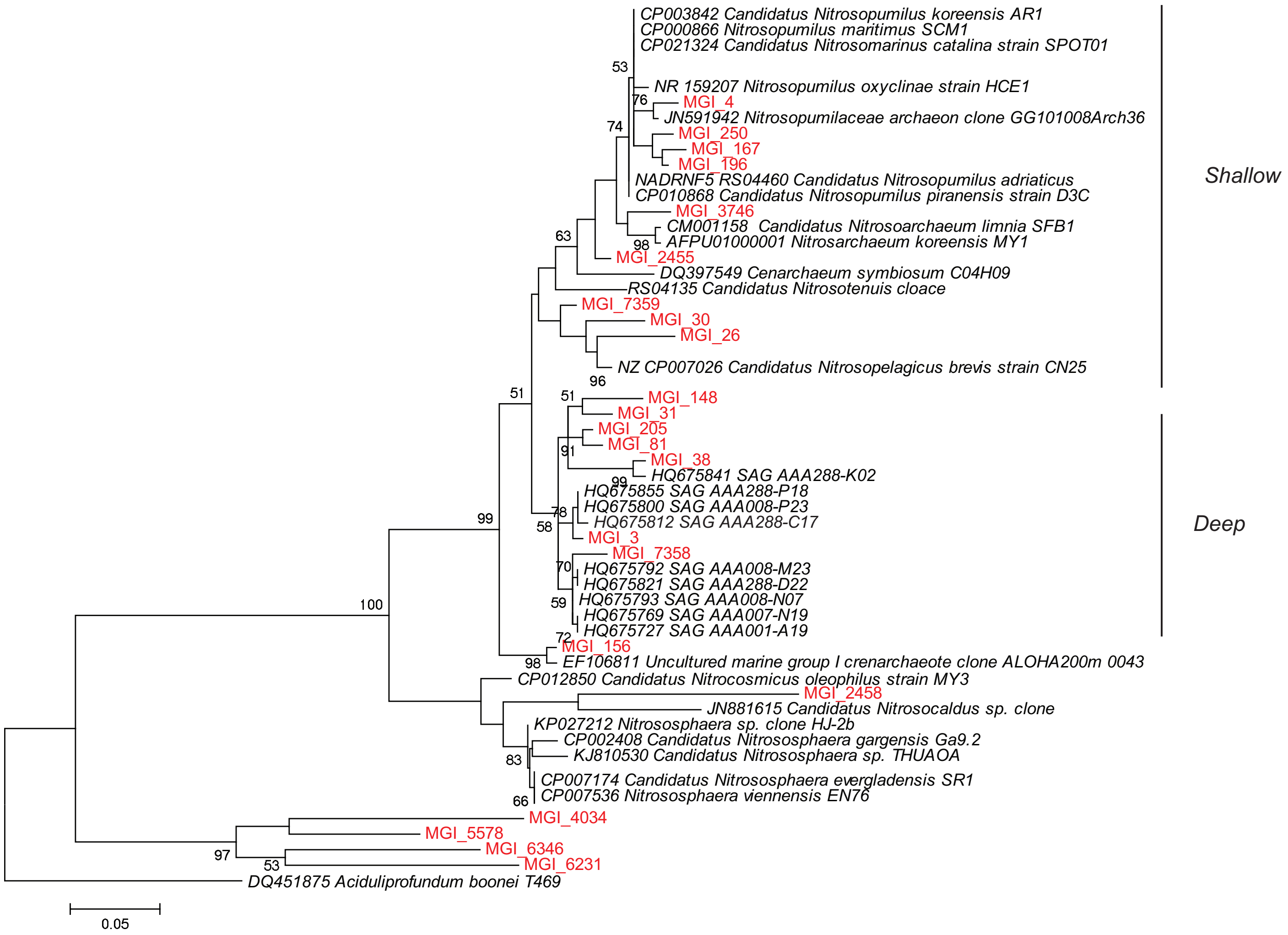

Figure S3. Top 20 most abundant archaeal OTUs *in situ* and during incubation as assessed by 16S rRNA sequencing of seawater from (A) Pacifica and (B) SPOT. Data is rescaled from Figure 2 to better demonstrate the stability of the archaeal community composition. Each bar represents a single sample or biological replicate (technical replicates were averaged). Red text and bars indicate incubations amended with bacterial antibiotics. Relative abundance out of total community. Colors are shared between sites. Asterisks indicate samples that were also analyzed via nanoSIMS.

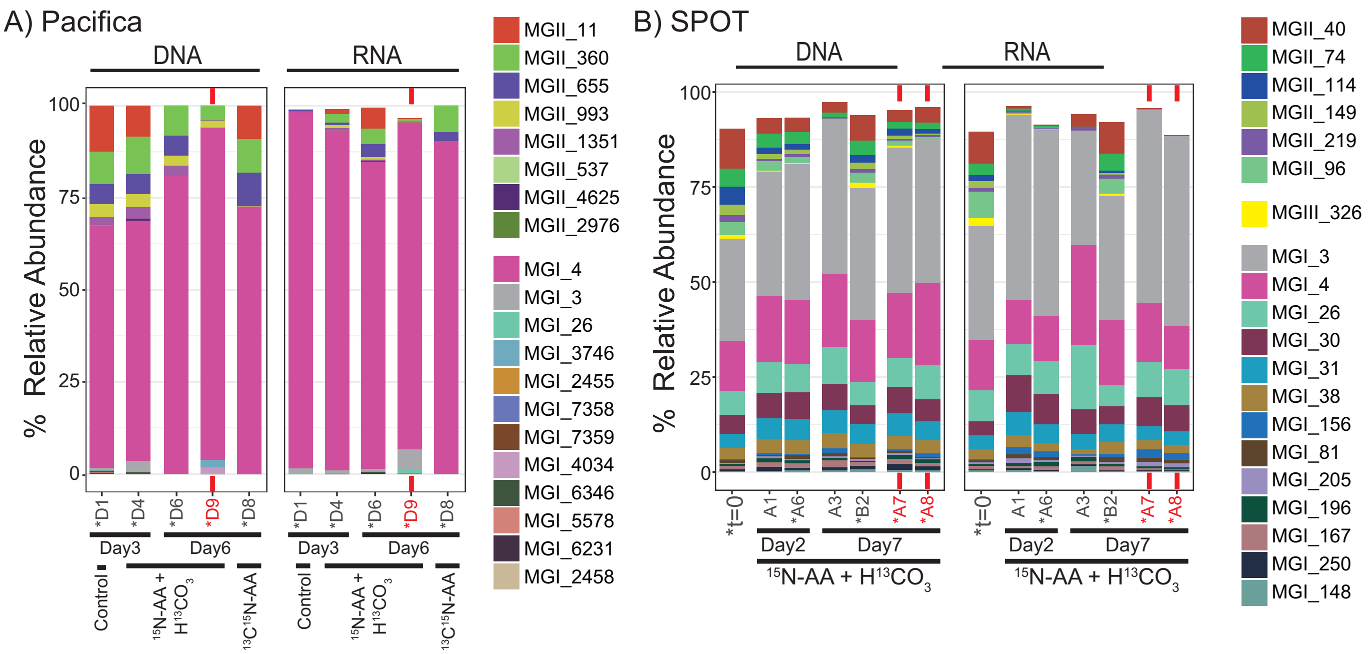

Figure S4. OTU-level changes in community composition over time and between treatments. Relative abundance of the top 50 most abundant OTUs and NMDS ordination of the Bray-Curtis Dissimilarity of the microbial communities *in situ* and during incubation as assessed by 16S rRNA sequencing of seawater from (A,B) Pacifica and (C,D) SPOT are shown. Bray-Curtis Dissimilarity was calculated using all OTUs in a sample. The number adjacent to each symbol in the NMDS plots indicates the day that sample was taken.

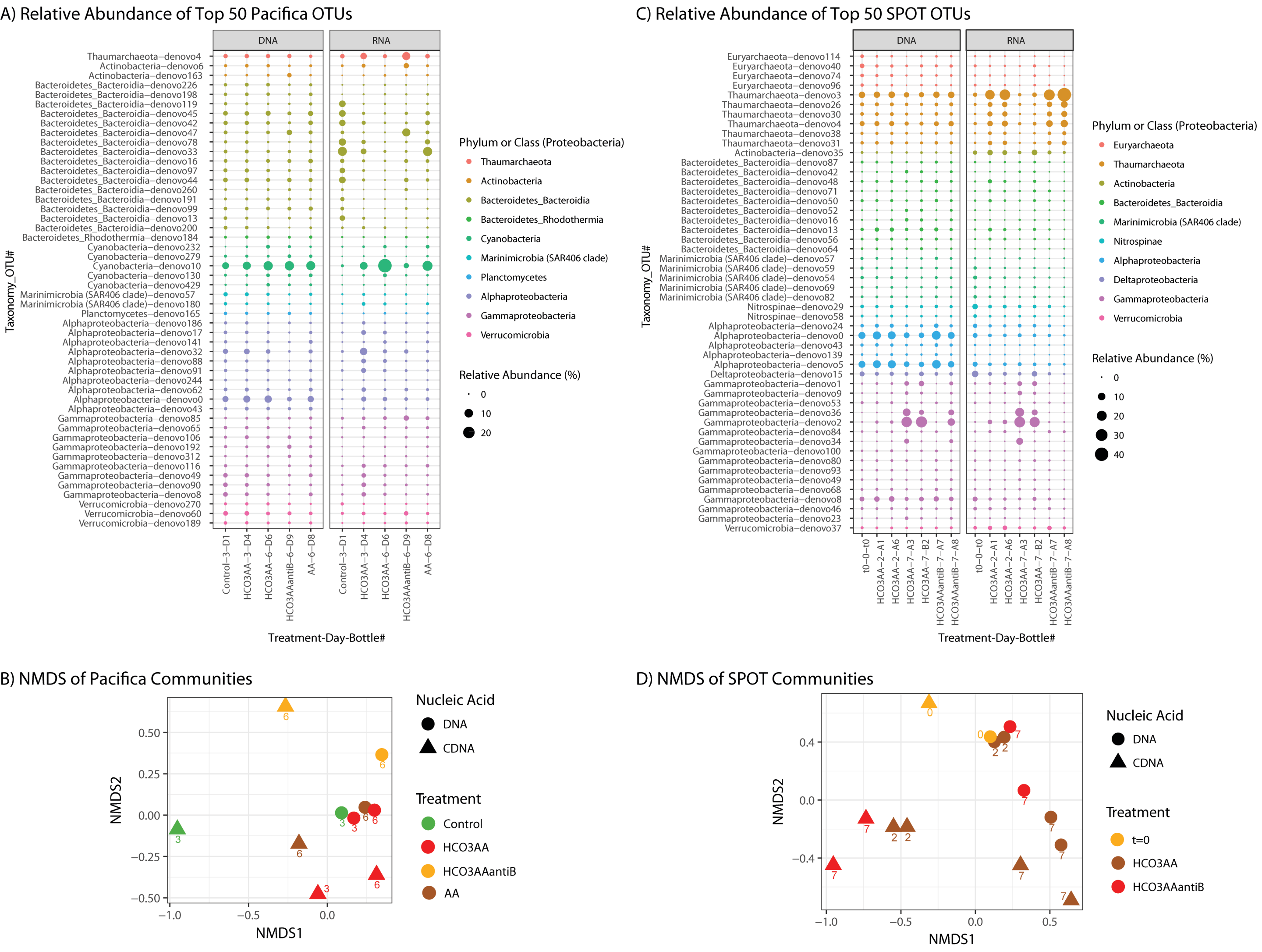

Figure S5. Archaeal community composition *in situ* and during incubation as assessed by 16S rRNA sequencing separated by the three major archaeal groups detected: MGI Thaumarchaeota (A+D), MGII Euryarchaeota (B+E) and MGIII Euryarchaeota (C+F), at both Pacifica (A-C) and SPOT (D-F). Shown as relative abundance of archaeal OTUs. The top 20 most abundant OTUs are shown for MGI and MGII, and the top 10 for MGIII. Each color represents a unique OTU.

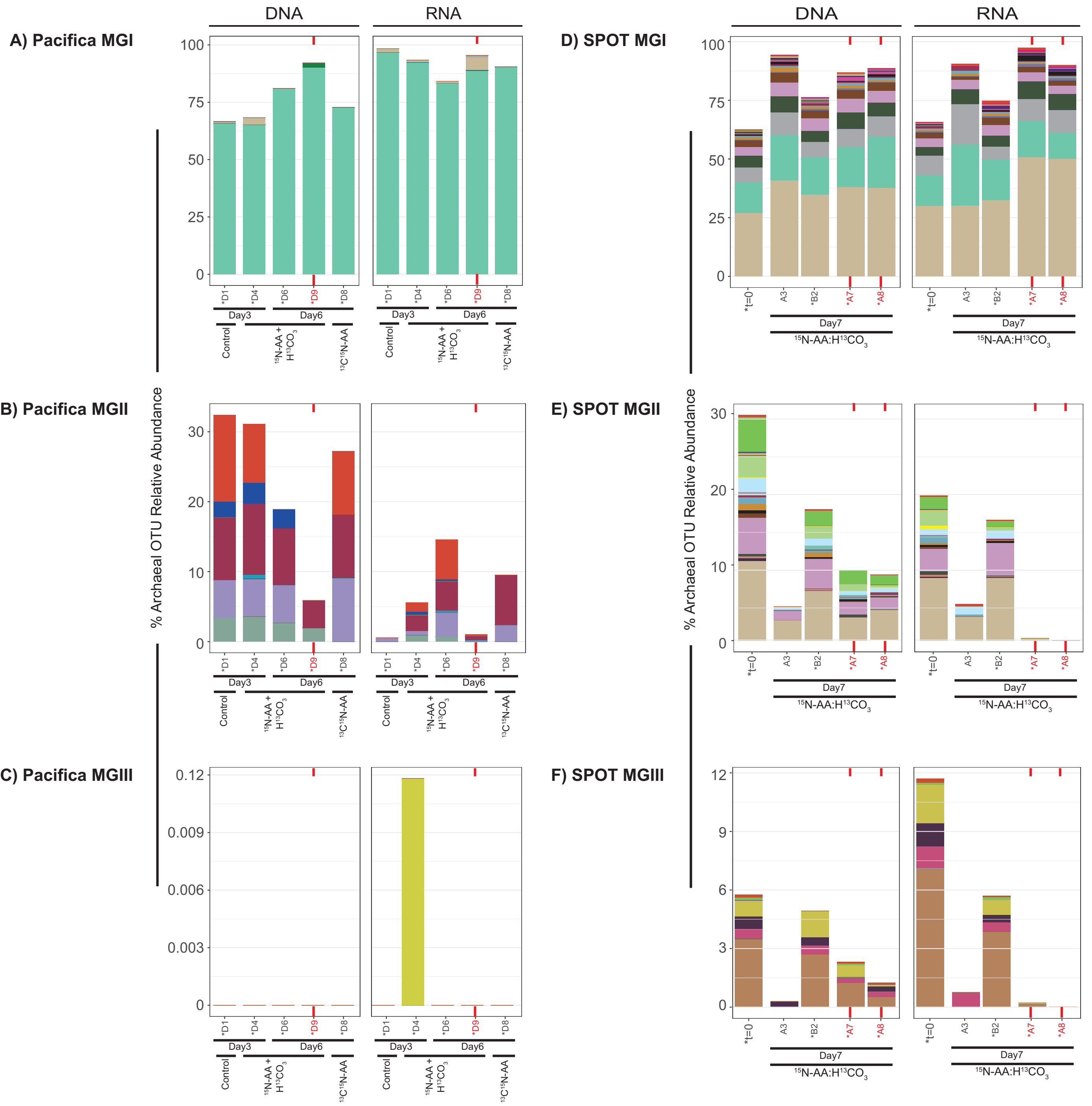

Supplement: Supplementary file 1 [file Data_Sheet_1.PDF]
